# Surgical Timing, Fracture Complexity and Healthcare-System Factors in Operatively Managed Distal Radius Fractures: A Retrospective Cohort Study

**DOI:** 10.3390/healthcare14213692

**Published:** 2026-11-01

**Authors:** Muhammad Umer Rasool, Muhammad Anas Ghazi, Mohammad Redwan, Sadaf Naderi, Muhammad Sohaib Khalid, Zain Habib, Basharat Ghafoor Khan, Otilia Manta, Kohila Vani Sigamoney

**Affiliations:** 1 Department of Trauma and Orthopaedics, North Manchester General Hospital, Manchester M8 5RB, UK; muhammadanas.ghazi@mft.nhs.uk (M.A.G.); mohammad.redwan@mft.nhs.uk (M.R.); sadaf.naderi@mft.nhs.uk (S.N.); m.khalid2@mft.nhs.uk (M.S.K.); kohila.sigamoney2@mft.nhs.uk (K.V.S.); 2 Department of Trauma and Orthopaedics, Bradford Teaching Hospitals NHS Foundation Trust, Bradford BD9 6RJ, UK; zain.habib@bthft.nhs.uk; 3 Department of Trauma and Orthopaedics, East Lancashire Hospitals NHS Trust, Blackburn BB2 3HH, UK; basharat.khan.tareen@gmail.com; 4 Romanian Academy, Centre for Financial and Monetary Research “Victor Slăvescu”, 050711 Bucharest, Romania; otilia.manta@rgic.ro; 5 Research Department, Romanian-American University, 012101 Bucharest, Romania

**Keywords:** distal radius fracture, surgical timing, trauma theatre capacity, healthcare delivery, healthcare utilisation, AO/OTA classification, complications, reoperation

## Abstract

**Highlights:**

**What are the main findings?**

- Service/capacity-related factors were documented in 52.8% of operatively managed distal radius fractures and were associated with substantially longer waits for surgery.
- Increasing injury-to-surgery interval was not significantly associated with postoperative complications, after adjustment for age and fracture complexity.

**What are the implications of the main findings?**

- Trauma-theatre capacity represents a potentially modifiable component of the distal radius fracture pathway.
- Prospective evaluation of pathway delays should incorporate clinical outcomes together with healthcare utilisation and economic consequences.

**Abstract:**

**Background/Objectives:** Surgical timing after distal radius fracture remains debated, while healthcare-system contributors to delay are less well described. We evaluated associations between time to surgery, fracture complexity, healthcare-pathway factors and postoperative complications. **Methods:** This retrospective single-centre cohort study used data from a registered clinical audit at North Manchester General Hospital. Patients aged ≥16 years undergoing operative management of an acute distal radius fracture between 18 March 2024 and 13 May 2025 were included. The primary outcome was any documented postoperative complication. Logistic regression assessed injury-to-surgery interval while adjusting for age and AO/OTA fracture complexity. **Results:** Eighty-nine patients were included; mean age was 57.9 years, and 74.2% were female. Median injury-to-surgery interval was 16 days (IQR 7–22) and did not differ across AO/OTA A, B and C groups (*p* = 0.582). Service/capacity factors were documented in 47/89 patients (52.8%) and were associated with longer waits than cases with no documented delay (median 18 versus 7.5 days; *p* < 0.001). Thirteen patients experienced complications (14.6%) and six underwent reoperation (6.7%). Each additional seven days to surgery was not significantly associated with complications, after adjustment (aOR 1.25, 95% CI 0.84–1.87; *p* = 0.269). **Conclusions:** Documented service/capacity-related factors were common and associated with longer observed surgical intervals. No statistically significant association was detected between broad AO/OTA type and surgical timing or between timing and documented postoperative complications. These findings do not establish causation or equivalence between earlier and later surgery. Larger prospective studies should evaluate clinical prioritisation, pathway factors and patient-centred outcomes.

## 1. Introduction

Distal radius fractures are among the most common adult orthopaedic injuries and create a substantial burden for emergency, trauma, and hand services. UK epidemiological data confirm their high incidence, and population-level studies show that the absolute number of fractures and the proportion treated surgically have increased over time [1,2]. Although many distal radius fractures can be managed non-operatively, unstable, displaced and articular injuries frequently undergo surgical fixation. Volar locking plate fixation is now the dominant operative technique in many health systems. Still, complications including nerve symptoms, complex regional pain syndrome, tendon irritation and hardware-related symptoms remain clinically important, and some patients subsequently require reoperation [3,4].

The optimal timing of surgical fixation remains uncertain. A 2023 systematic literature review found marked heterogeneity in how surgical delay was defined and concluded that the evidence linking time to surgery with clinical and radiographic outcomes was conflicting [5]. A separate systematic review using a two-week threshold suggested that delays beyond two weeks may be associated with worse patient-reported disability. However, range of motion, grip strength, radiographic outcomes and pooled complication rates were broadly similar between early and delayed groups [6]. UK data have also reported a lack of clinically important long-term difference between timely and delayed surgery in a large retrospective cohort [7].

Other studies indicate that delay may nevertheless influence operative difficulty, early recovery, and healthcare utilisation. Luangjarmekorn et al. reported longer operative times, more frequent bone grafting, and a greater need for additional incisions when fixation was delayed beyond 10 days [8]. Liu et al. found that surgery more than 14 days after injury was associated with greater clinic and hand-therapy utilisation and worse early wrist flexion [9]. Population-level data have further suggested increased reoperation when fixation occurs beyond three weeks after failed closed reduction [10], while a large 2025 study identified the lowest complication rates in an intermediate 6–15-day window rather than immediately after injury [11]. Most recently, a 2026 systematic review and meta-analysis of 14 studies involving 2104 patients reported a higher complication rate with later surgery, while patient-reported outcome measures did not differ significantly [12]. Taken together, the literature still leaves a practical question unanswered: which outcomes are most sensitive to delay, and at what point does waiting become clinically important?

Fracture complexity may confound the relationship between timing and outcome. Complete articular AO/OTA type C fractures have been associated with increased postoperative complication risk in large observational cohorts [13,14]. However, the AO/OTA classification is more reproducible at the broad A/B/C level than at the subgroup level, supporting the use of broad fracture-type categories for clinical analysis [15].

The healthcare system also influences timing. Delays can arise from medical optimisation, surgical planning, patient factors, referral pathways, surgeon availability, and trauma theatre capacity. UK multicentre data from the ORTHOPOD programme demonstrated substantial variation in day-case trauma pathways, limited dedicated trauma-list capacity and longer waits for some day-case injuries, highlighting the relevance of organisational factors to surgical access [16]. These determinants are less often incorporated into distal radius fracture outcome studies than patient or fracture characteristics.

The primary aim of this study was to evaluate the association between injury-to-surgery interval and postoperative complications among patients undergoing operative management of acute distal radius fractures. The secondary aims were to determine whether AO/OTA fracture complexity was associated with surgical timing or complications, to characterise documented healthcare-, clinical- and patient-related factors associated with surgical timing, and to describe postoperative complication and reoperation patterns.

## 2. Materials and Methods

### 2.1. Study Design and Setting

This retrospective single-centre cohort study used data collected as part of a registered clinical audit at North Manchester General Hospital, a district general hospital in the United Kingdom. The cohort comprised patients undergoing surgery between 18 March 2024 and 13 May 2025. Injury could precede the start of this operative inclusion window. The analysis was designed and reported in accordance with the Strengthening the Reporting of Observational Studies in Epidemiology (STROBE) recommendations for cohort studies [17].

### 2.2. Study Population

Patients aged 16 years or older who underwent operative fixation of an acute distal radius fracture during the operative inclusion period were eligible—operative treatment comprised open reduction and internal fixation (ORIF) or percutaneous K-wire fixation. Patients with open fractures not requiring additional procedures for soft-tissue coverage, those with an associated ulna fracture, and those transferred or referred from other hospitals were included. Patients undergoing delayed reconstructive surgery for established distal radius malunion, including corrective osteotomy, were excluded because these procedures represented a different clinical pathway from acute fracture fixation. We excluded fractures managed entirely non-operatively and duplicate clinical episodes. After applying the eligibility criteria, 89 patients formed the final cohort.

### 2.3. Data Collection

We retrospectively extracted data from routinely collected electronic clinical records, operative documentation, and radiographic records. Variables included date of birth, sex, injury date, presentation date, surgery date, AO/OTA fracture classification, operative treatment, documented factor associated with surgical delay, postoperative complications, reoperation and reoperation procedure. Age was calculated from date of birth to date of surgery. Injury-to-surgery, presentation-to-surgery, and injury-to-presentation intervals were calculated in days.

We did not include patient-reported outcome measures, range-of-motion measurements, or smoking status because they were not required to address the specified research objectives and were incompletely documented.

### 2.4. Fracture Classification

Fractures were classified according to the AO/OTA system by two clinicians reviewing preoperative, intraoperative and postoperative radiographs, with operative findings considered where available. Disagreements were discussed with an experienced clinician. Following peer-review feedback, a clinician reassessed radiographs for all 89 patients and reclassified nine extra-articular fractures from A3 to A2. Detailed subtypes are reported descriptively; inferential analyses used broad A, B and C groups. The multivariable model compared type C against A/B to limit model complexity [15].

### 2.5. Classification of Factors Associated with Surgical Timing

Documented reasons associated with surgical timing were categorised from clinical records and communications involving treating clinicians and trauma coordinators. Service/capacity factors included trauma theatre capacity, theatre cancellation, surgeon availability and referral or transfer delays. Clinical/planning factors included medical optimisation, investigations and operative planning. Patient/presentation factors included delayed presentation, initial reluctance to undergo surgery and unavailability for proposed dates owing to personal commitments. Loss of fracture position was classified as fracture evolution; fracture-clinic pathway delays were classified as pathway/follow-up factors. Cases explicitly documented as having no delay were retained separately. These retrospective categories do not establish causation.

### 2.6. Outcomes

The primary outcome was any documented postoperative complication, comprising metalwork- or screw-related symptoms, complex regional pain syndrome, neurological symptoms or compression, and fixation failure. The four categories were mutually exclusive and each affected patient was counted once. Stiffness was excluded because objective range-of-motion and validated patient-reported outcome measurements were not consistently available. Reoperation was a secondary descriptive outcome. Complications and reoperations were ascertained from available electronic clinical records, postoperative reviews and subsequent intervention records. Follow-up duration was defined as the interval from index surgery to the last documented postoperative review; follow-up was not standardised.

### 2.7. Statistical Analysis

We summarised continuous variables using mean and standard deviation when appropriate, and median and interquartile range (IQR) for non-normally distributed data. Categorical variables were reported as frequencies and percentages. We evaluated differences in injury-to-surgery intervals across AO/OTA groups and documented delay categories using the Kruskal–Wallis test. We used the Mann–Whitney U test for selected two-group comparisons, including surgical timing in patients with and without complications and comparisons between patients with service/capacity-related factors and those with no documented delay. Associations between categorical variables were evaluated using the chi-square test or Fisher’s exact test, as appropriate.

An exploratory multivariable logistic regression model evaluated the association between injury-to-surgery interval (per seven days) and any documented postoperative complication, adjusting for age and broad AO/OTA fracture classification (type C versus A/B). Only 13 patients experienced the outcome, giving approximately 4.3 events per predictor before accounting for the intercept. Accordingly, maximum-likelihood estimates may be unstable, and the model was interpreted as exploratory rather than confirmatory. Firth bias-reduced penalised logistic regression was additionally fitted as a sensitivity analysis with the same three predictors. Penalised profile-likelihood 95% confidence intervals and likelihood-ratio *p*-values were calculated. This analysis was exploratory and was not part of a prospectively registered statistical analysis plan. The separate 14-day comparison was also exploratory.

### 2.8. Ethics and Governance

The project was registered and conducted as a clinical audit at North Manchester General Hospital (audit reference: 13027). It retrospectively analysed routinely collected anonymised clinical records without altering patient care. Under applicable local clinical audit governance arrangements, formal Research Ethics Committee approval and individual consent were not required. Audit registration should not be construed as prospective registration of a detailed statistical analysis plan.

### 2.9. Use of Generative Artificial Intelligence

ChatGPT (OpenAI; GPT-5.6 Sol).) was used as an assistive tool during data analysis to help check statistical calculations, organise and interpret analytical output, and review the clarity of statistical reporting. It was also used to improve the English language use, grammar, consistency and manuscript formatting. The authors retained responsibility for the data extraction, clinical classification, selection and verification of analyses, interpretation of findings and approval of the final manuscript. AI-generated suggestions were reviewed by the authors and were not accepted as independent evidence or as a substitute for checking against the study data. The exact author-approved AI disclosure is reproduced in the Acknowledgements.

## 3. Results

### 3.1. Cohort Characteristics

**Figure 1:**
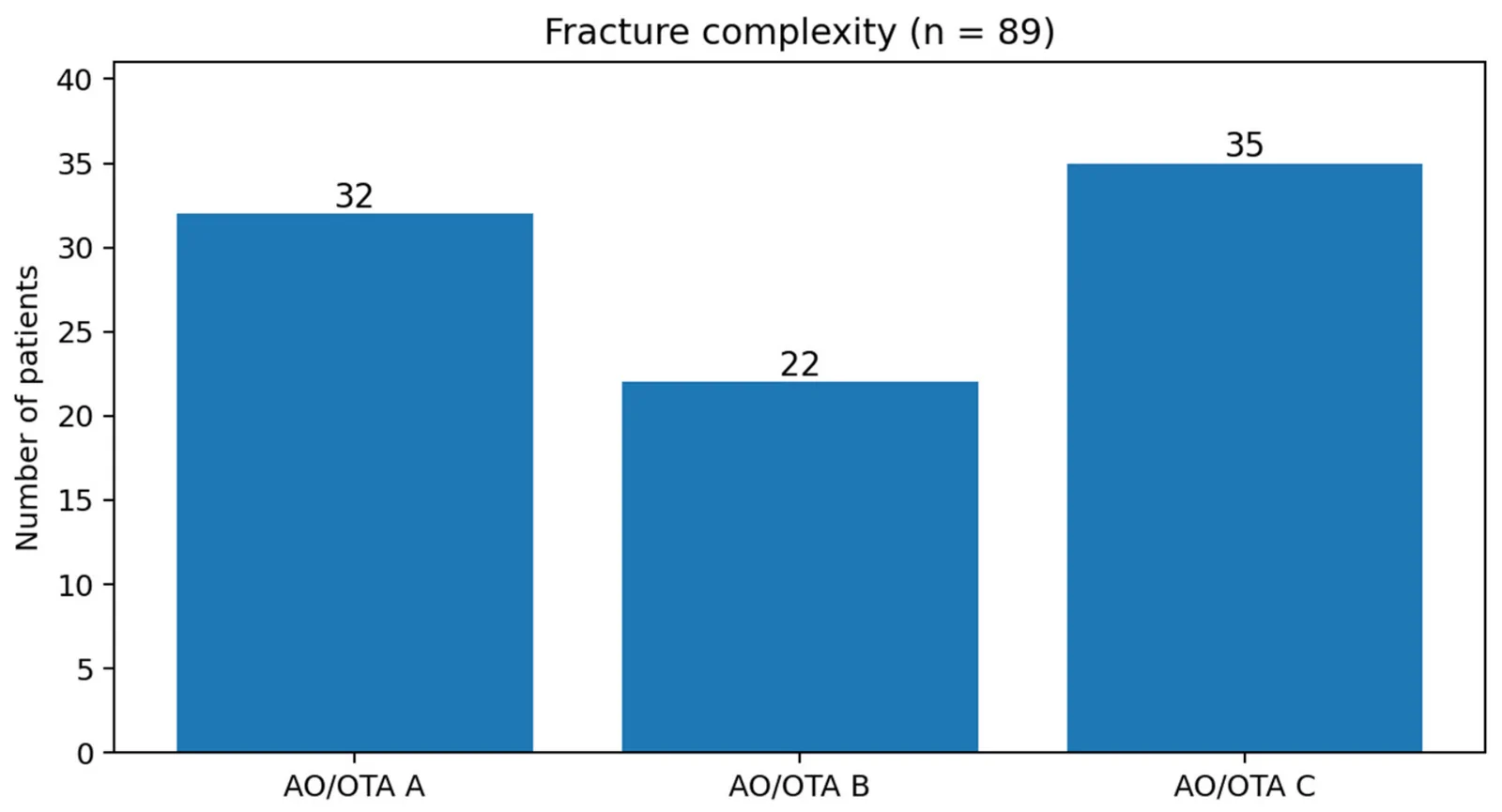
Distribution of broad AO/OTA fracture groups (*n* = 89).

The cohort comprised 89 patients (mean age 57.9 ± 17.0 years; 66 female, 74.2%). AO/OTA types A, B and C accounted for 32 (36.0%), 22 (24.7%) and 35 (39.3%) patients, respectively (Figure 1). Following reassessment of all 89 radiographs by a clinician, the detailed classification was A2: 9, A3: 23, B1: 1, B2: 2, B3: 19, C1: 19, C2: 11 and C3: 5. Four patients (4.5%) had open fractures with puncture wounds, none requiring soft-tissue coverage. Associated ulna injuries were limited to ulnar styloid fractures, none requiring fixation. All 89 underwent fixation of the distal radius only (ORIF 84; K-wire fixation 5). Median injury-to-surgery interval was 16 days (IQR 7–22), presentation-to-surgery 11 days (IQR 5–21), and injury-to-presentation 0 days (IQR 0–1) (Table 1).

### 3.2. Fracture Complexity and Surgical Timing

Median injury-to-surgery interval was 17 days (IQR 10.75–22) for AO type A fractures, 10.5 days (IQR 7–22.5) for type B fractures and 16 days (IQR 6.5–22) for type C fractures. There was no statistically significant difference in timing across AO/OTA groups (Kruskal–Wallis *p* = 0.582) (Table 2).

### 3.3. Documented Factors Associated with Surgical Timing

Service/capacity-related factors were documented in 47 patients (52.8%), clinical/planning factors in 17 (19.1%), patient/presentation factors in three (3.4%), fracture evolution in one (1.1%), and pathway/follow-up factors in one (1.1%). Twenty patients (22.5%) were explicitly documented as having no delay. Trauma theatre-list capacity accounted for 45 of the 47 service/capacity-related cases.

Patients with documented service/capacity factors had a median injury-to-surgery interval of 18 days (IQR 10–22.5), compared with 7.5 days (IQR 3.5–15.3) in the no-documented-delay group (Mann–Whitney *p* < 0.001). Presentation-to-surgery interval was also longer (median 13 versus 7.5 days; *p* = 0.044). Service/capacity factors occurred in 17/32 (53.1%) type A, 14/22 (63.6%) type B and 16/35 (45.7%) type C fractures (*p* = 0.418). Observational comparisons remain susceptible to clinical prioritisation and residual confounding.

### 3.4. Postoperative Complications and Reoperation

Complication status was available for all 89 patients (Table 3). Thirteen distinct patients had a documented complication (14.6%; 95% CI 8.7–23.4%): metalwork- or screw-related symptoms in nine, complex regional pain syndrome in one, neurological symptoms or compression in two, and fixation failure in one. The categories were mutually exclusive. Six patients underwent reoperation (6.7%), all with a documented complication. The indications were metalwork removal in two, combined median nerve compression at the carpal tunnel and ulnar nerve compression at Guyon’s canal in one, fixation failure in one, and metalwork irritation with median nerve compression at the carpal tunnel in two.

Complications occurred in 5/32 (15.6%) type A fractures, 2/22 (9.1%) type B fractures and 6/35 (17.1%) type C fractures (*p* = 0.689). The A2/A3 correction did not alter the 32 type A cases or the broad-group complication analysis. The five complications within type A are reported at the broad-group level used for the primary analyses; no separate A2-versus-A3 complication comparison was undertaken, because of sparse subgroup events.

### 3.5. Follow-Up Duration

Median recorded postoperative follow-up was 3.0 months (IQR 1.5–5.0). Median follow-up was 2.8 months in patients undergoing surgery within 14 days, and 3.0 months among those undergoing surgery after 14 days (*p* = 0.303). Follow-up was not standardised, and these summary figures describe recorded follow-up rather than complete long-term surveillance.

### 3.6. Surgical Timing and Postoperative Complications

Patients with a postoperative complication had a median injury-to-surgery interval of 19 days (IQR 8–27), compared with 15.5 days (IQR 7–22) among those without a documented complication (Mann–Whitney *p* = 0.262). In the primary multivariable model, each additional seven days from injury to surgery was associated with an adjusted OR for postoperative complication of 1.25 (95% CI 0.84–1.87; *p* = 0.269). Age was not significantly associated with complication risk (adjusted OR 1.02 per year, 95% CI 0.98–1.07; *p* = 0.232), nor was AO/OTA type C fracture compared with A/B (adjusted OR 1.35, 95% CI 0.40–4.54; *p* = 0.624) (Table 4).

In the 14-day sensitivity analysis, complications occurred in 5/40 (12.5%) patients undergoing surgery within 14 days and 8/49 (16.3%) undergoing surgery after 14 days (Fisher exact *p* = 0.765). After adjustment for age and AO/OTA complexity, surgery after 14 days was not significantly associated with postoperative complications (adjusted OR 1.27, 95% CI 0.37–4.33; *p* = 0.699).

Firth bias-reduced penalised logistic regression was additionally performed as an exploratory sensitivity analysis. Bias-reduced estimation yielded similar effect estimates: injury-to-surgery interval per seven days (adjusted OR 1.23, 95% CI 0.84–1.82; *p* = 0.280), age per year (adjusted OR 1.02, 95% CI 0.99–1.07; *p* = 0.224), and AO/OTA type C versus A/B (adjusted OR 1.37, 95% CI 0.42–4.38; *p* = 0.597). These estimates were consistent in direction and magnitude with the primary analysis, but the confidence intervals remained wide.

### 3.7. Exploratory Analysis of Service/Capacity Factors and Complications

Complications occurred in 7/47 (14.9%) patients with a service/capacity-related factor and 6/42 (14.3%) patients without one. The unadjusted OR was 1.05 (95% CI 0.32–3.42; Fisher exact *p* = 1.000). In an exploratory model adjusted for age and AO/OTA complexity, the adjusted OR was 1.04 (95% CI 0.31–3.45; *p* = 0.952). These analyses were considered exploratory because event numbers were limited.

## 4. Discussion

### 4.1. Principal Findings

In this retrospective cohort, the median injury-to-surgery interval was 16 days. Documented service/capacity-related factors were frequent and associated with longer observed waiting times, but the study cannot establish whether these factors caused delay independently of clinical prioritisation. Broad AO/OTA group was not significantly associated with timing. The adjusted association between waiting time and recorded postoperative complications was imprecise and not statistically significant; these results should not be interpreted as evidence of equivalent safety between early and delayed fixation.

### 4.2. Surgical Timing and Outcomes

NICE guideline NG38 and BOAST 16 recommend fixation within 72 h of injury for intra-articular distal radius fractures and within seven days for extra-articular fractures; for redisplacement following manipulation, the recommended interval is within 72 h of the decision to operate [18,19]. The median injury-to-surgery interval of 16 days in our cohort exceeds these timeframes as an overall descriptive statistic. However, the cohort includes delayed presentations, initial non-operative management with subsequent loss of position, referrals/transfers, and other clinical or service factors; an injury-based median alone cannot establish patient-level guideline adherence. Our study was not designed as a formal NICE/BOAST compliance audit, and we therefore do not report an overall compliance percentage. These findings nonetheless support closer prospective measurement of the decision-to-operate date, indication-specific targets and potentially modifiable capacity constraints. The literature does not point to a single, simple timing threshold. Julian et al. [5] found substantial variation in how delay and outcomes were defined across 15 studies and concluded that the effect of timing remained uncertain. Khan et al. [6] used a two-week threshold and reported worse frequency-weighted Disabilities of the Arm, Shoulder and Hand (DASH) scores after delayed surgery, but a similar range of motion, grip strength, radiographic outcomes, and low complication and revision rates. In a UK cohort of 380 patients, Howard et al. [7] found no clinically or statistically significant long-term difference between timely and delayed groups. This inconsistency matters when interpreting our results: a non-significant association in one cohort does not establish that timing is unimportant.

Timing may also matter in ways that a binary complication outcome does not capture. Fixation beyond 10 days has been associated with longer operating time, more bone graft use, and additional incisions [8], while surgery after 14 days has been associated with greater clinic and hand-therapy use and worse early wrist flexion [9]. In a population study of 14,960 patients, Nowak et al. [10] found higher reoperation odds when fixation occurred more than 21 days after failed closed reduction. Persitz et al. [11] subsequently reported the lowest observed complication and infection rates in an approximately 6–15-day window, raising the possibility that the relationship between timing and adverse outcomes is non-linear.

Evidence regarding the clinical implications of surgical timing remains heterogeneous. In the cited 2026 systematic review and meta-analysis, Kotze et al. [12] reported an association between later surgery and a higher complication risk. Our exploratory adjusted point estimate (OR 1.25 per additional week; 95% CI 0.84–1.87) points in the same direction but is imprecise and includes no association. With only 13 events and three model predictors, sparse-data bias and overfitting remain important concerns. Firth bias-reduced penalised regression was therefore performed as an exploratory sensitivity analysis using the same three predictors. The resulting estimates were similar to those from the conventional model, although confidence intervals remained wide and clinically important effects cannot be excluded. Our data therefore neither refute previous evidence of harm from delay nor demonstrate that delay is safe.

### 4.3. Fracture Complexity

The relationship between fracture complexity and complications varies across the literature. Thorninger et al. found a significant association between AO/OTA type C fractures and complications in 576 patients [13], and Li et al. identified type C morphology as an independent risk factor in 1175 fractures [14]. In our cohort, complication proportions did not differ significantly across types A, B and C. A further 822-patient series by Perregaard et al. likewise found no association between fracture type or time from trauma to surgery and postoperative complications [20]. Differences in sample size, case mix, follow-up and complication definitions probably account for some of this variation. Our use of broad A/B/C categories rather than sparse subgroups is supported by reliability work showing substantially better reproducibility at the AO type level than at the group or subgroup level [15].

### 4.4. Healthcare-System Factors

Documented service/capacity factors were recorded in 47 of 89 patients, with a median injury-to-surgery interval of 18 days, compared with 7.5 days among those without a documented delay. Presentation-to-surgery interval was also longer in the service/capacity group. The retrospective data cannot distinguish the effects of theatre capacity from clinical prioritisation, referral pathways and residual confounding. No statistically significant association was detected between broad AO/OTA type and service/capacity documentation; this does not exclude an influence of fracture characteristics on prioritisation.

This pattern fits with wider UK orthopaedic trauma evidence. ORTHOPOD found marked variation in day-case trauma pathways, limited dedicated day-case trauma-list provision and substantial cancellation pressure across participating centres [16]. Our study adds a patient-level distal radius perspective: when a capacity problem was documented, the observed wait was materially longer. A UK service evaluation by Assafiri et al. [21] found that introduction of a dedicated day-case hand centre was associated with improved same-day discharge and reduced length of stay; this supports further investigation of dedicated pathways but does not establish that such a pathway would reduce injury-to-surgery time in our cohort.

Complication proportions were 14.9% in patients with documented service/capacity-related factors and 14.3% in those without such factors. These descriptive proportions must not be interpreted as demonstrating equivalent outcomes: the comparison is underpowered, the groups may differ in clinical prioritisation, and complications were ascertained through non-standardised follow-up.

### 4.5. Multidisciplinary Relevance

The pathway is inherently multidisciplinary. Orthopaedic teams decide whether and how to fix the fracture; anaesthetic and perioperative teams determine whether medical optimisation is needed; theatre managers and trauma coordinators influence access to operating capacity; and hand therapists manage part of the downstream recovery burden. Our findings are therefore directly relevant to at least these disciplines. In particular, separating clinically necessary postponement from capacity-related postponement gives teams a more useful target for service improvement than treating every extra day to surgery as equivalent. Prior evidence that delayed surgery may increase early therapy and clinic use also suggests that scheduling decisions can shift workload beyond the operating theatre [9].

### 4.6. Healthcare-Resource, Health-Economic and Prevention Implications

The documented association between service/capacity factors and longer waiting intervals suggests a potentially important service-delivery question, but no causal or financial effect can be quantified from this dataset. We did not collect treatment costs, length-of-stay costs, therapy utilisation or productivity losses. The previous literature on delayed fixation and resource use may provide context, but extrapolating a monetary saving from the present findings would be inappropriate. Prospective studies should measure clinical urgency, the date of decision to operate, cancellations, theatre capacity, patient outcomes and resource utilisation together.

The financial implications nevertheless require careful interpretation. Economic evaluations of distal radius fracture treatment demonstrate that higher-resource interventions are not necessarily more cost-effective. UK trial-based analyses have shown higher treatment costs for volar locking-plate fixation than K-wire fixation, with relatively small differences in health benefit [22,23], while other trial-based economic evaluations indicate that cost-effectiveness varies according to patient characteristics, treatment strategy and societal factors such as return to work [24]. Therefore, the economic implication of our findings is not that earlier surgery will necessarily reduce overall costs, but that potentially modifiable capacity-related delays represent an appropriate target for service evaluation. Prospective studies incorporating theatre costs, length of stay, repeat clinic attendance, rehabilitation and hand-therapy use, reoperation, time away from work and quality-adjusted life-years would be required to determine whether reducing such delays translates into measurable financial benefit for healthcare systems.

Beyond the timing of surgery, prevention is also relevant when considering the wider burden of distal radius fractures on healthcare services. Many of these injuries occur in the context of fragility, particularly in older adults, providing an opportunity to identify and address potentially modifiable factors such as risk of falls and underlying osteoporosis. Secondary prevention pathways, including fracture liaison services, have been associated with a reduced risk of subsequent fragility fractures and may also be cost-effective [25,26]. From a wider healthcare perspective, preventing further fractures could reduce future demand for emergency care, surgery, outpatient follow-up and rehabilitation, while timely and appropriate management of established fractures may help make better use of available healthcare resources. These considerations are particularly relevant where operative capacity is limited. However, healthcare costs and resource utilisation were not directly evaluated in our study, and these potential benefits should therefore be viewed as broader implications rather than conclusions drawn from the present cohort.

### 4.7. Strengths and Limitations

This study describes fracture classification, surgical timing, documented pathway factors and clinical outcomes at patient level. The broad AO/OTA groups used for inferential analyses were unchanged following clinician reassessment of all 89 radiographs, which corrected nine subtype classifications from A3 to A2. The analysis of timing as a continuous exposure avoids relying exclusively on an arbitrary binary threshold, although the study remains observational and exploratory.

The limitations include the retrospective single-centre design, a modest sample size and only 13 documented complication events. The three-predictor conventional logistic regression has approximately 4.3 events per predictor and is susceptible to sparse-data bias, overfitting and wide confidence intervals. Firth bias-reduced penalised logistic regression was performed as a sensitivity analysis and yielded similar point estimates; nevertheless, penalisation does not resolve the limited number of events, wide confidence intervals, unmeasured confounding or potential outcome under-ascertainment. Delay categories relied on routine records and may be incomplete or misclassified, while clinical prioritisation and referral pathways may confound associations. The median 16-day injury-to-surgery interval should not be interpreted as a uniform waiting time or a stand-alone measure of guideline compliance. Median documented follow-up was three months, but surveillance was not standardised; late complications may have been missed. Postoperative stiffness was deliberately excluded from the predefined primary complication outcome because objective range-of-motion assessments and validated patient-reported measures were inconsistently recorded; subjective stiffness entries were not considered sufficiently standardised for a clinically interpretable stiffness-inclusive sensitivity model. This exclusion may underestimate overall postoperative morbidity and precludes conclusions about stiffness outcomes. Open fractures, ulnar styloid fractures and referred or transferred patients introduced clinical heterogeneity. The five K-wire cases precluded meaningful fixation-specific comparisons. Functional outcomes, healthcare costs and resource utilisation were not systematically measured.

### 4.8. Implications for Practice and Research

Future prospective multicentre work should capture the decision-to-operate date, fracture-specific urgency, transfer and referral processes, theatre availability, cancellations and reasons for postponement. Standardised follow-up, validated patient-reported outcomes and consistent complication definitions would permit more reliable comparisons. Larger event counts would support adequately powered adjusted modelling and prespecified bias-reduced sensitivity analyses. Formal evaluation of healthcare utilisation and costs is needed before concluding that pathway changes improve economic outcomes.

## 5. Conclusions

In this retrospective cohort of 89 operatively managed distal radius fractures, documented service/capacity factors, predominantly trauma theatre-list availability, were common and associated with longer observed surgical intervals. No statistically significant association was detected between broad AO/OTA type and timing or between timing and recorded postoperative complications. Given the sparse events, non-standardised follow-up and potential confounding, these findings do not establish causality or equivalence between earlier and later surgery. Larger prospective multicentre studies should evaluate clinical prioritisation, service constraints, functional outcomes and healthcare-resource implications.

## Figures and Tables

**Table 1:** Demographic, fracture and operative characteristics of the study cohort.

Characteristic	Result
Total cohort	89
Age, mean ± SD, years	57.9 ± 17.0
Age, median (range), years	61.9 (16.6–88.4)
Female sex	66 (74.2%)
Male sex	23 (25.8%)
AO/OTA type A	32 (36.0%)
AO/OTA type B	22 (24.7%)
AO/OTA type C	35 (39.3%)
A2	9 (10.1%)
A3	23 (25.8%)
B1	1 (1.1%)
B2	2 (2.2%)
B3	19 (21.3%)
C1	19 (21.3%)
C2	11 (12.4%)
C3	5 (5.6%)
Open reduction and internal fixation	84 (94.4%)
K-wire fixation	5 (5.6%)
Injury-to-surgery interval, median (IQR), days	16 (7–22)
Presentation-to-surgery interval, median (IQR), days	11 (5–21)
Injury-to-presentation interval, median (IQR), days	0 (0–1)
Postoperative follow-up, median (IQR), months	3.0 (1.5–5.0)
Open fractures (puncture wounds; no coverage required)	4 (4.5%)
Associated ulna injuries requiring fixation	0

Data are n (%) unless otherwise specified. IQR, interquartile range; SD, standard deviation.

**Table 2:** Surgical timing and documented factors associated with delay.

Group	n (%)	Injury-to-Surgery, Median (IQR), Days	Statistical Comparison
AO/OTA type A	32	17 (10.75–22)	Kruskal–Wallis *p* = 0.582
AO/OTA type B	22	10.5 (7–22.5)	
AO/OTA type C	35	16 (6.5–22)	
Service/capacity-related	47 (52.8%)	18 (10–22.5)	vs. no delay: *p* < 0.001
No documented delay	20 (22.5%)	7.5 (3.5–15.25)	
Clinical/planning-related	17 (19.1%)	-	
Patient/presentation-related	3 (3.4%)	-	
Fracture evolution	1 (1.1%)	-	
Pathway/follow-up-related	1 (1.1%)	-	

Presentation-to-surgery interval was longer in the service/capacity group than in the no-documented-delay group (median 13 versus 7.5 days; *p* = 0.044). Trauma theatre-list capacity accounted for 45 of 47 service/capacity cases. Sparse delay categories were described without emphasising the omnibus test.

**Table 3:** Postoperative outcomes and complication categories.

Outcome	Result
Any documented postoperative complication	13/89 (14.6%)
No documented postoperative complication	76/89 (85.4%)
Reoperation	6/89 (6.7%)
Metalwork/screw-related symptoms	9
Complex regional pain syndrome	1
Neurological symptoms/compression	2
Failed fixation	1

The four complication categories were mutually exclusive and comprised 13 distinct patients. Stiffness was excluded from the primary outcome definition because objective assessments were inconsistent. The absence of a documented complication should be interpreted as no recorded complication rather than confirmation that no complication occurred.

**Table 4:** Multivariable logistic regression predictors of postoperative complications.

Predictor	Adjusted OR	95% CI	*p*-Value
Time to surgery, per additional 7 days	1.25	0.84–1.87	0.269
Age, per year	1.02	0.98–1.07	0.232
AO/OTA type C vs. A/B	1.35	0.40–4.54	0.624

Primary logistic regression outcome: any documented postoperative complication, with postoperative stiffness excluded. Sensitivity analysis for surgery >14 days versus ≤14 days: adjusted OR 1.27 (95% CI 0.37–4.33; *p* = 0.699).

## Data Availability

The dataset contains clinical audit data derived from hospital records and is not publicly available, because of institutional governance and confidentiality requirements. The corresponding author may provide an anonymised data extract upon reasonable request, subject to approval by the relevant institutional data-governance authority.

## Abbreviations

The following abbreviations are used in this manuscript:

AO/OTA	Arbeitsgemeinschaft für Osteosynthesefragen/Orthopaedic Trauma Association
CI	Confidence interval
CRPS	Complex regional pain syndrome
DASH	Disabilities of the Arm, Shoulder and Hand
IQR	Interquartile range
NHS	National Health Service
OR	Odds ratio
ORIF	Open reduction and internal fixation
STROBE	Strengthening the Reporting of Observational Studies in Epidemiology
